# The Feasibility of Large Language Models in Verbal Comprehension Assessment: Mixed Methods Feasibility Study

**DOI:** 10.2196/68347

**Published:** 2025-02-24

**Authors:** Dorit Hadar-Shoval, Maya Lvovsky, Kfir Asraf, Yoav Shimoni, Zohar Elyoseph

**Affiliations:** 1 Department of Psychology Max Stern Academic College of Emek Yezreel Afula Israel; 2 School of Psychology University of Haifa Haifa Israel; 3 Department of Education and Psychology Open University of Israel Raanana Israel; 4 Department of Brain Sciences Faculty of Medicine Imperial College London London United Kingdom; 5 School of Counseling and Human Development Faculty of Education University of Haifa Haifa Israel

**Keywords:** large language models, verbal comprehension assessment, artificial intelligence, AI in psychodiagnostics, personalized intelligence tests, verbal comprehension index, Wechsler Adult Intelligence Scale, WAIS-III, psychological test validity, ethics in computerized cognitive assessment

## Abstract

**Background:**

Cognitive assessment is an important component of applied psychology, but limited access and high costs make these evaluations challenging.

**Objective:**

This study aimed to examine the feasibility of using large language models (LLMs) to create personalized artificial intelligence–based verbal comprehension tests (AI-BVCTs) for assessing verbal intelligence, in contrast with traditional assessment methods based on standardized norms.

**Methods:**

We used a within-participants design, comparing scores obtained from AI-BVCTs with those from the Wechsler Adult Intelligence Scale (WAIS-III) verbal comprehension index (VCI). In total, 8 Hebrew-speaking participants completed both the VCI and AI-BVCT, the latter being generated using the LLM Claude.

**Results:**

The concordance correlation coefficient (CCC) demonstrated strong agreement between AI-BVCT and VCI scores (Claude: CCC=.75, 90% CI 0.266-0.933; GPT-4: CCC=.73, 90% CI 0.170-0.935). Pearson correlations further supported these findings, showing strong associations between VCI and AI-BVCT scores (Claude: *r*=.84, *P*<.001; GPT-4: *r*=.77, *P*=.02). No statistically significant differences were found between AI-BVCT and VCI scores (*P*>.05).

**Conclusions:**

These findings support the potential of LLMs to assess verbal intelligence. The study attests to the promise of AI-based cognitive tests in increasing the accessibility and affordability of assessment processes, enabling personalized testing. The research also raises ethical concerns regarding privacy and overreliance on AI in clinical work. Further research with larger and more diverse samples is needed to establish the validity and reliability of this approach and develop more accurate scoring procedures.

## Introduction

Cognitive assessment is one of the core skills of applied psychologists, and it is considered one of the most important contributions of scientific psychology to clinical practice [1]. Intelligence tests are widely used to measure individuals’ cognitive abilities. They provide a standardized measure of a person’s intellectual capacities, aiding in educational placement and career selection, and identifying potential cognitive impairments. Intellectual assessment measures serve as valuable tools in psychology, education, and research, helping identify gifted individuals, track cognitive development, and evaluate the effectiveness of interventions [1]. Intelligence tests have their limitations [2] but their consistent use and refinement over time have contributed to our understanding of human intelligence and its variations. Traditionally, the development of an intelligence test involves an intricate process and the construction of age- and gender-based norms based on extensive data collected from a sizable sample representative of the population [3].

Intelligence tests are designed to assess various cognitive abilities, such as logical reasoning, problem-solving skills, spatial awareness, and verbal comprehension [4]. The verbal comprehension index (VCI) is known for its strong correlation with the g factor (the hypothesized construct of general intelligence) [5], and it measures the capability to use language for reasoning, comprehension, and understanding social norms. The VCI includes three subtests, that are, similarities, vocabulary, and information [5].

The significance of a comprehensive psychological assessment including measures of verbal intelligence cannot be overstated. [6-10]. The assessment of verbal intelligence also extends to personality traits crucial for interpersonal interactions, such as the recognition of emotions and the ability to express oneself effectively [11]. The comprehensive psychological evaluation of various populations under different conditions requires a nuanced understanding of verbal intelligence.

The administration of intelligence tests and the subsequent interpretation of the results are typically undertaken by psychologists. These professionals usually possess at least a master’s degree in psychology or in a related field, which qualifies them to conduct the tests and analyze the results accurately and ethically. Intellectual testing is usually administered in a standardized form, where the individuals being tested are presented with similar tasks under similar conditions. The standardized administration process helps ensure an ethical process and produces valid results, leading to accurate interpretations [1,10].

The field of intelligence testing still heavily relies on conventional paper-and-pencil tests, despite recent efforts to embrace technological advancements. Some current intelligence testing measures, such as the fifth edition of the Wechsler Intelligence Scale for Children [12], presuppose the use of tablets by clinicians to improve the administration and scoring procedures. Globally, however, the use of technology in psychological assessment is still developing and to date has had relatively modest influence.

At the end of 2022, Open AI launched ChatGPT-3.5, a generative artificial intelligence (GenAI) product based on a large language model (LLM) trained on abundant text data and capable of generating human-like responses to text-based inputs [13]. This open access application set a record as the fastest-growing consumer application in history [14], making significant contributions to academia [13], psychology [14-27], medicine [28], and programming [29]. In March 2023, Anthropic launched Claude, another GenAI product based on LLM, with a similar interface to that of ChatGPT but with Hebrew language capabilities that are considered to be more nuanced and accurate than those of ChatGPT.

While these technological advances offer promising opportunities for psychological assessment, it is essential to acknowledge their current limitations. LLMs cannot replace comprehensive clinical evaluations, as they lack the ability to observe crucial behavioral cues, interpret complex clinical contexts, or make nuanced professional judgments. Therefore, any application of AI in cognitive testing must be viewed as a potential supportive tool requiring professional oversight.

This pilot study represents an innovative endeavor in the field of GenAI and its application in psychological assessment, serving as a proof-of-concept investigation into the feasibility of using LLMs for verbal comprehension testing. Rather than aiming to validate these tools across a large population, our primary objective was to demonstrate whether LLMs can, in principle, generate personalized verbal comprehension assessments that produce results comparable to standardized tests. Central to this study is the development of a personalized cognitive test comprising 30 questions without human norms (artificial intelligence [AI]–based verbal comprehension test [AI-BVCT]). Unlike standard testing procedures, this approach does not rely on pre-established diagnostic tools but uses the algorithmic capabilities of the LLM to encode, assess, and calculate verbal intelligence scores.

A critical aspect of this study is the comparative analysis conducted between the AI-BVCT results and those obtained from the VCI, under the supervision of an expert psychologist. To establish the basic viability of this approach, we conducted a focused comparison between AI-generated tests and traditional assessments on a small sample. We hypothesized that the differences between the AI-BVCT and traditional VCI scores would be within half an (SD 7.5), indicating a high level of accuracy and that the correlation between the AI-BVCT and the VCI would be high, demonstrating accurate cognitive ranking across participants. By focusing exclusively on verbal comprehension abilities, the study aims to establish proof of concept for how LLMs can contribute to psychological testing, emphasizing the potential for new personalized and dynamic testing methodologies.

## Methods

### Overview

This study used 2 LLMs: GPT-4 (OpenAI) and Claude (Anthropic). Data collection occurred in September 2023. The selection of these models was based on several key considerations. At the time of the study, these models represented the most advanced capabilities in natural language processing, particularly for the Hebrew language. Claude demonstrated superior proficiency in Hebrew language processing and a nuanced understanding of cultural context, making it our primary choice for generating the AI-BVCT. Both models were used to evaluate participants' responses, allowing for a comparison between different approaches to language model development. The use of models from different leading companies (OpenAI and Anthropic) enabled us to examine various approaches to language model development and training, providing a broader context for our findings. It is important to note that this selection limits the study to Western commercial models and does not include open-source models or those developed in other cultural contexts. This limitation will be discussed further in the paper. We did not alter their default settings when using these models. We used the standard chat interface provided by the companies, without special adjustments to hyperparameters such as temperature or top-k. An additional limitation of these tools is their ownership by commercial entities, which restricts full transparency of their training and development processes. It is also worth noting that the field of LLMs is rapidly evolving, and more advanced models have become available since the study was conducted.

### Participants

This pilot study included 8 native Hebrew-speaking volunteer participants (4 females), aged 28.5 (SD 2.13) years. The participants were recruited through random sampling, facilitated by a research assistant who distributed targeted recruitment notices to undergraduate and graduate psychology students at Max Stern Yezreel Valley College. The recruitment process extended over a 2-month period, during which participants volunteered without compensation. The recruitment notices specifically outlined the study’s objectives and requirements, ensuring informed participation. All participants who initially enrolled completed the study in its entirety, with no withdrawals recorded during the research period. The participants were selected from the student population due to their accessibility and their presumed average to above-average cognitive abilities, which were deemed appropriate for this pilot study. However, we acknowledge the limitations of this small, nonrepresentative sample, which are addressed in detail in the limitations section of this paper.

### Research Design and Procedure

The study used a within-participants design, with each participant undergoing 2 cognitive tests: the VCI from the WAIS-III, and a personalized AI-BVCT generated by the LLM Claude system.

After each participant underwent the VCI test, the participant’s age and gender were entered into the LLM Claude system, together with a prompt to generate a personalized AI-BVCT to measure the participant’s verbal intelligence. In the next stage, the participant was required to respond to the AI-BVCT. The participant’s responses were then fed into 2 separate LLMs, Claude and GPT-4, with a prompt containing scoring instructions. To ensure the reliability of the scoring across different runs, each participant’s responses were entered into each of the 2 models 10 times, using a separate tab for each iteration. As a result, each participant received a total of 20 scores (10 from each model) on the AI-BVCT.

**Figure 1:**
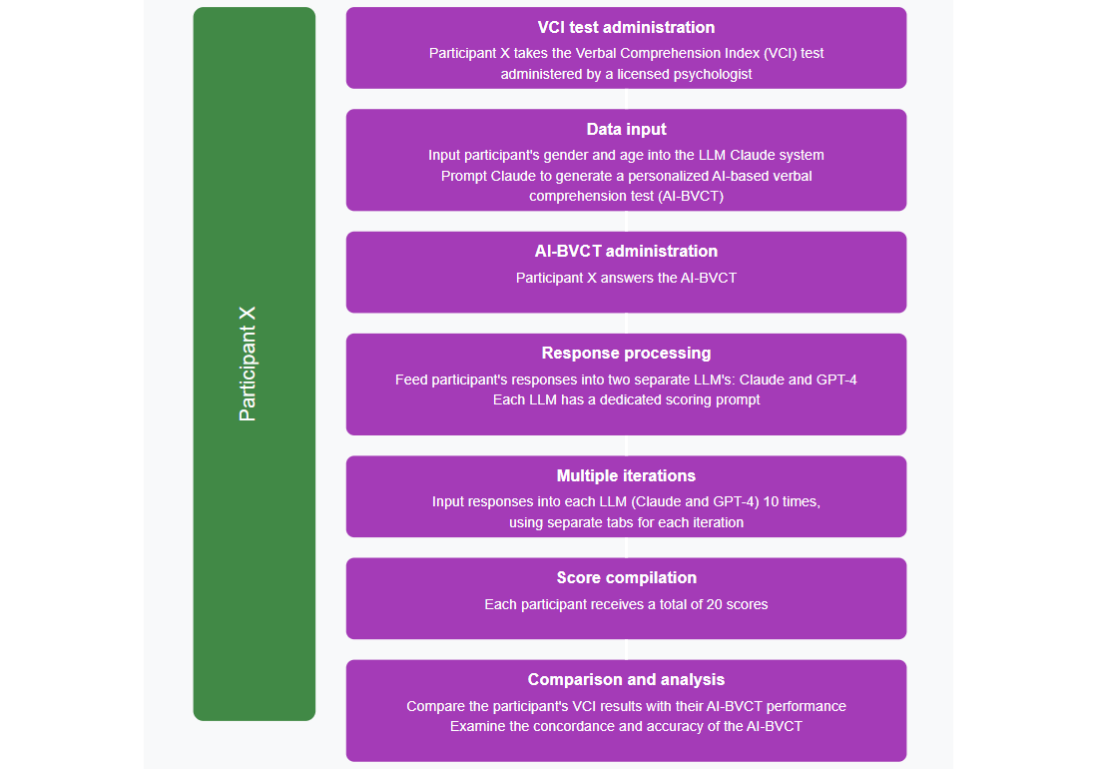
Schematic representation of the study procedure for assessing verbal comprehension using AI-based tests in a proof-of-concept study of Hebrew-speaking university students (N=8) in Israel, September 2023. AI-BVCT: artificial intelligence-based verbal comprehension tests; LLM: large language models; VCI: verbal comprehension index.

Finally, we compared the participant’s results on the VCI test and their performance on the AI-BVCT to examine the degree of concordance and accuracy of the test developed by the LLM system. A schematic representation of the study procedure is provided in Figure 1.

### Materials

#### The VCI

##### Overview

The VCI is an index designed to assess verbal abilities, such as verbal concept formation and reasoning skills. This index is part of the Wechsler Adult Intelligence Scale-3 (WAIS-III; [30]), which measures various cognitive abilities in adults. VCI scores are normalized with a mean of 100 and an SD of 15 [31]. The WAIS-III was used in this study as it represents the most recent version of the WAIS-III that has been officially translated, adapted, and normalized for Hebrew-speaking populations in Israel. While newer versions exist, they have not yet been standardized for Hebrew-speaking populations.

The index consists of three subtests: (1) similarities, which measures the ability to identify commonalities between terms; (2) vocabulary, which measures the understanding and use of words; and (3) information, which measures general knowledge and the ability to provide factual information [5]. The VCI score is calculated by combining the 3 subtests.

##### VCI Scoring

Scoring was performed according to the accepted scoring manual. Each question was assigned a score of 0-2 based on the acceptable responses provided in the scoring guide. The calculation of standard scores and results was conducted in accordance with the guidelines established in the WAIS manual.

#### Personalized AI-Based Verbal Comprehension Test

##### Generating the Personalized AI-BVCT

The LLM Claude (Anthropic, Ltd) was used for generating a personalized cognitive test for each participant. We used Claude because at the time the study was conducted its performance in Hebrew was considered superior to those of other LLMs, such as GPT-4 or Bard. To generate the AI-BVCT, we composed a request for Claude to develop a 30-item verbal intelligence test, having provided the participant’s age and gender. The inclusion of age information was intended to ensure age-appropriate content and difficulty level, similar to standardized tests. Gender information was provided to account for the gendered nature of the Hebrew language in question phrasing, although the content and difficulty of questions were not intended to differ based on gender. To enhance the quality of the test, we also provided examples of questions to guide Claude in shaping questions with the appropriate structure and difficulty level. Specifically, we provided 3 sample items for each of the 3 intelligence levels, which consist of low (<70), medium (90-110), and high (>125) for each of the subtests.

##### Personalized AI-BVCT Prompt

The prompt that was entered into Claude are shown in Textbox 1.

**Prompt for constructing a verbal intelligence test with artificial intelligence.**

Cognitive Assessment

- Act as a professor of psychology specializing in psychodiagnostics and psychometrics. Please create a 30-question test that can assess the verbal intelligence score of an X-year-old Hebrew-speaking man or woman. Try to create questions whose answers will be as similar as possible to the results of the verbal intelligence WAIS 3 test in the verbal intelligence index, which includes a vocabulary test (defining words at increasingly difficult levels), common denominator (finding a semantic common denominator between words at increasingly difficult levels), and general knowledge (general knowledge at increasingly difficult levels). Construct the questions so that they can give as accurate a score as possible in the range of 50-150. This means that some questions should be very easy and some very difficult. Build questions that can be answered via the chat box, without any human intervention, and without questions requiring additional tools other than the chat itself.

To refine your ability to build questions at different difficulty levels, you are given examples.

- Vocabulary: (1) Here are some words for which the level of difficulty to define for a 30-year-old adult is hard and reflects a higher than average verbal intelligence (verbal intelligence level 125 and above): obelisk, orthography, ether, allegory, atom. (2) Here are some words for which the level of difficulty to define for a 30-year-old adult is medium and reflects average verbal intelligence (verbal intelligence level 90-110): fortification, settlement, takeover, degeneration, outsmarting. (3) Here are some words for which the level of difficulty to define for a 30-year-old adult is easy and reflects much lower-than-average verbal intelligence (verbal intelligence level 70 and below: table, chair, cupboard, sofa, television
- SimList Paragraphilarities: (1) Here are some words for which the level of difficulty to find a common denominator for a 30-year-old adult is hard and reflects higher than average verbal intelligence (verbal intelligence level 125 and above): clothes and food, dream and reality, life and death, light and darkness, sky and earth. (2) Here are some words for which the level of difficulty to find a common denominator for a 30-year-old adult is medium and reflects average verbal intelligence (verbal intelligence level 90-110): wave and wind, freedom and imprisonment, land and sea, inside and outside, number and letter. (3) Here are some words for which the level of difficulty to find a common denominator for a 30-year-old adult is easy and reflects much lower than average verbal intelligence (verbal intelligence level 70 and below). Fork and knife, ball and football, rain and snow, dog and cat, pants and shirt.
- General Knowledge: (1) Here are some questions that reflect high general knowledge of a 30-year-old adult reflecting higher than average verbal intelligence (verbal intelligence level 125 and above): Who painted “The Scream”? Who wrote the Odyssey? In which century did Beethoven live? In which sea are the Maldives Islands located? What is the biological component that converts RNA to protein? (2) Here are some questions that reflect the medium general knowledge of a 30-year-old adult reflecting average verbal intelligence (verbal intelligence level 90-110): Who was the first king of Israel according to the Bible? Who invented the telephone? In which country is the famous Himalayan Mountain, Everest, located? In which year did World War II end? Who was Nelson Mandela? (3) Here are some questions that reflect the low general knowledge of a 30-year-old adult reflecting much lower than average verbal intelligence (verbal intelligence level 70 and below): What is the vehicle that moves over water and carries people? On which Israeli holiday is it customary to light candles? What animal is the king of animals? What is the name of the first month of the Gregorian calendar? What shape do most balls have?
- Now build the test to assess verbal intelligence. The examples provided to you are meant to reflect different levels of difficulty to help you build a test sensitive to a score range of 50-150. You don't need to build the questions in the same structure as the examples (common denominator, vocabulary, and knowledge).

##### The AI-BVCT Scoring

After the participants completed the AI-BVCT, their responses to the test questions were entered into 2 LLMs, Claude and GPT-4, for evaluation and scoring. The LLMs were instructed to review and grade the participants’ responses to each of the 30 questions, assigning a score ranging from 0 to 2 for each answer (0=incorrect, 1=partial answer, and 2=full answer), making the total possible score range 0-60. Given the assumption that the test has a normal distribution of difficulty levels, correctly answering half the test questions (15 correct answers score of 30) is considered equivalent to a verbal IQ score of 100, correctly answering all test questions (30 correct answers, a raw score of 60) indicates a verbal IQ score of 150. Consequently, each point above or below 30 in the AI-BVCT corresponds to an IQ change of 1.67 points. For example, the IQ score of participants whose AI-BVCT was determined to be 31 would be 101.67. To increase reliability and avoid random errors, each participant’s responses were entered into each of the LLM 10 times.

The scoring process sought to address 2 main challenges. The first is that the LLMs struggle to perform numeric summation consistently. The second concerns certain cases where the LLMs incorrectly classify a correct response as incorrect or an incorrect response as correct. To address these challenges, we took two steps: (1) an MA student in psychology manually scored each participant’s responses to the AI-BVCT and (2) the automatic scoring process was supervised by the researchers regarding two key criteria in Textbox 2.

**Correction criteria for artificial intelligence–based scoring errors.**

- In cases where there was an error in summing the number of correct responses into a total score [For example, in a case where a participant correctly answered 10 questions (2 points each) and partially answered 10 questions (1 point each), the large language model (LLM) erroneously calculated a total score of 29 instead of the correct 30], the LLMs were instructed: “You have summed the cases incorrectly, please re-sum.”
- In extreme cases where the LLMs assigned a score of 2 when the human scorer assigned a score of 0, or vice versa [For example, when asked to define “ambivalence,” a participant responded: “Ambivalence is the simultaneous existence of conflicting attitudes or emotions towards an object or situation.” The human rater scored this as fully correct (2 points), but the LLM incorrectly assigned 0 points.], we instructed the LLMs: “You have made an error on question number X, classifying it as incorrect when it is correct. Please adjust the score accordingly.” While this correction process was necessary for this proof-of-concept study, we acknowledge that such iterative adjustments could potentially introduce bias. Future implementations should consider automated validation methods and more structured scoring protocols to minimize human intervention.

##### Prompt for Reviewing the Personalized AI-BVCT

The prompt used is outlined in Textbox 3.

**Prompt for reviewing and scoring artificial intelligence–based verbal comprehension test (AI-BVCT) responses.**

*(Insert the participant's responses to the test questions here)*

*Your task now is to create a table in the following order*

- In column 1, write the question and its number.
- In column 2, write the participant’s answer.
- In column 3, carefully review each question based on your knowledge. Cross-reference different information sources and score according to the following key: a correct answer receives a score of 2. A partial or not entirely accurate answer receives a score of 1. Also, give a score of 1 for a slight inaccuracy. An entirely incorrect answer or a statement from the participant that they do not know the answer receives a score of 0. Ignore spelling errors in the scoring.
- In column 4, indicate the cumulative score accumulated throughout the questions.
- Below the table, write how many correct and partially correct answers there were out of 30 and calculate the intermediate score by accurately summing the scores obtained in step c. Caution: Please ensure the sum is correct before submitting the answer.
- Perform an additional calculation, this time counting the number of errors separately and noting it.
- Verify that the number of correct, partially correct, and incorrect answers adds up correctly to a total of 30 questions.
- After verification, indicate the final intermediate score at the end-this should be the score calculated in section e.

##### Prompt for Scoring the Personalized AI-BVCT

The following prompts were used:

(Insert the intermediate score obtained from the AI-BVCT here)

Now you need to convert the obtained score to a new score. First, create a heading: “Final Score Calculation”

Calculation method: If the participant received an intermediate score of 30 by answering 15 questions correctly with 2 points each, they will receive a score of 100. For each point above the intermediate score of 30, add 1.67 points (a participant who received an intermediate score of 31 will receive a final score of 101.67). The same applies in reverse for scores below 15 correct answers; they will receive below 100 in a similar ratio.

### Ethical Considerations

This study was approved by the institutional review board of Max Stern Yezreel Valley College (approval number: YVC 2023-66). All participants provided written informed consent after receiving a comprehensive explanation of the research procedures and objectives. Data were collected anonymously and stored securely with access restricted to the research team. All identifying information was removed from responses before analysis. Participants received course credit as compensation for their participation. No personally identifiable information or images were included in the study materials or findings.

### Statistical Analysis

To assess the agreement between the AI-BVCTs and the VCI scores, we computed the intraclass correlation coefficient (ICC) and Bland-Altman limits of agreement (LoA; a method to assess agreement between 2 different measurement techniques by plotting the differences against the averages of the 2 measures). We also calculated the concordance correlation coefficient (CCC) to evaluate the agreement between the AI-BVCTs and the VCI scores (CCC measures the degree to which pairs of observations fall on the 45° line through the origin, indicating perfect agreement). We used Pearson correlations to examine the association between the VCI score, AI-BVCT Claude 2 scores, and AI-BVCT GPT-4 scores. The reliability of the AI-BVCTs was assessed using internal consistency (Cronbach α).

To test the hypothesis that the differences between the AI-BVCTs and VCI scores would be bounded within equivalence bounds of 15 points, we conducted Two One-Sided Tests (TOST) and paired-samples *t* test (TOST, used to establish statistical equivalence between 2 measures). We compared the proportion of AI-BVCT Claude 2 and GPT-4 scores falling within the 95% WAIS CI using a *z* test.

Finally, we used a generalized mixed model to investigate the differences between the AI-BVCT Claude 2 scores and GPT-4 scores and their interaction with the VCI scores. We conducted post hoc comparisons to examine the differences between the LLMs at various levels of VCI (mean, SD 1) using Jamovi (version 2.3.28).

## Results

### Preliminary Analysis

First, we examined the reliability of the 10 AI-BVCTs generated by each LLM. Both LLMs had high internal consistency (AI-BVCT Claude 2: α=.988, AI-BVCT GPT-4: α=.986). Next, we computed the ICC to assess the agreement between the AI-BVCTs and VCI scores. The ICC was 0.961 (95% CI 0.872-0.991; 2-way mixed, average measures, absolute agreement), which is considered excellent reliability [32].

**Figure 2:**
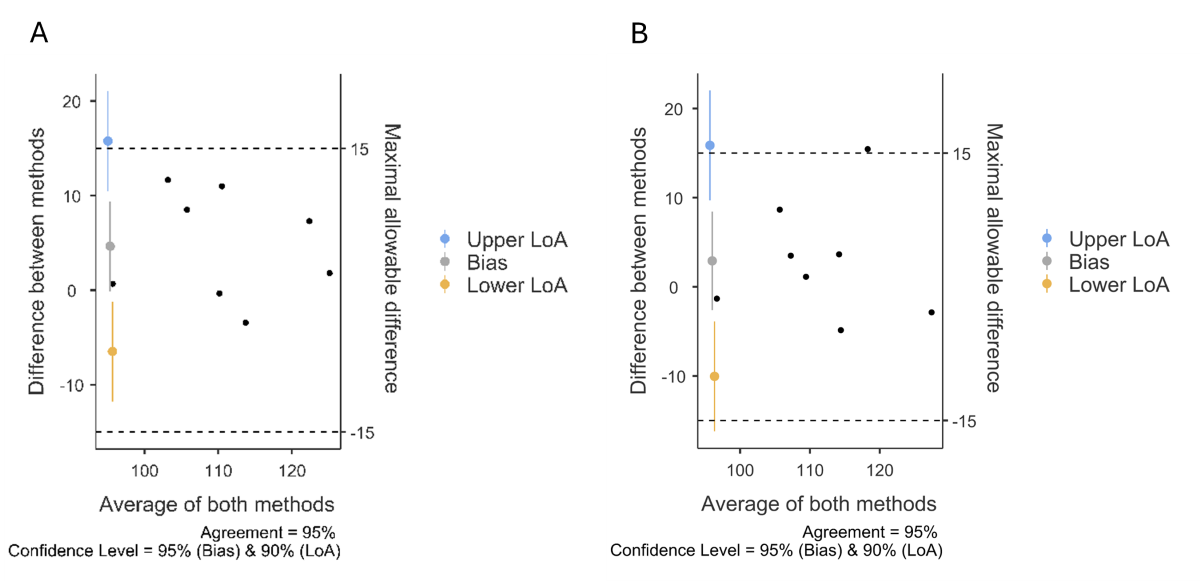
Bland-Altman plots demonstrating agreement between VCI scores and AI-generated verbal comprehension test scores in Hebrew-speaking university students (N=8): A comparison of traditional and AI-based assessment methods. Study conducted in Israel, September 2023. LoA: limits of agreement.

We then proceed to calculate Bland-Altman LoA (Figure 2) and the CCC.

For AI-BVCT Claude 2, the Bland-Altman LoA indicated that the mean bias (4.64) was not significantly different from 0 (95% CI –0.10 to 9.38), the lower LoA was –6.48 (90% CI –11.78 to 1.18), and the upper LoA was 15.76 (90% CI 10.47-21.06). The CCC was 0.752 (90% CI 0.266-0.933), which is considered an almost excellent agreement.

For AI-BVCT GPT-4, the Bland-Altman LoA indicated that the mean bias (2.91) was not significantly different from 0 (95% CI –2.61 to 8.43), the lower LoA was –10.04 (90% CI –16.21 to –3.87), and the upper LoA was 15.86 (90% CI 9.70-22.03). The CCC was 0.733 (90% CI 0.170-0.935), which is considered an almost excellent agreement [33].

Following these results, we found high Pearson correlations between the VCI score and AI-BVCT Claude 2 scores (*r*=0.844, *P*<.001), the VCI score and AI-BVCT GPT-4 scores (*r*=0.771, *P*=.02), and AI-BVCT Claude 2 and AI-BVCT GPT-4 scores (*r*=0.866, *P*=.01).

In sum, the verbal IQ-like scoring of the AI-BVCTs generated by the LLMs was found to be reliable.

### Hypothesis Testing

**Figure 3:**
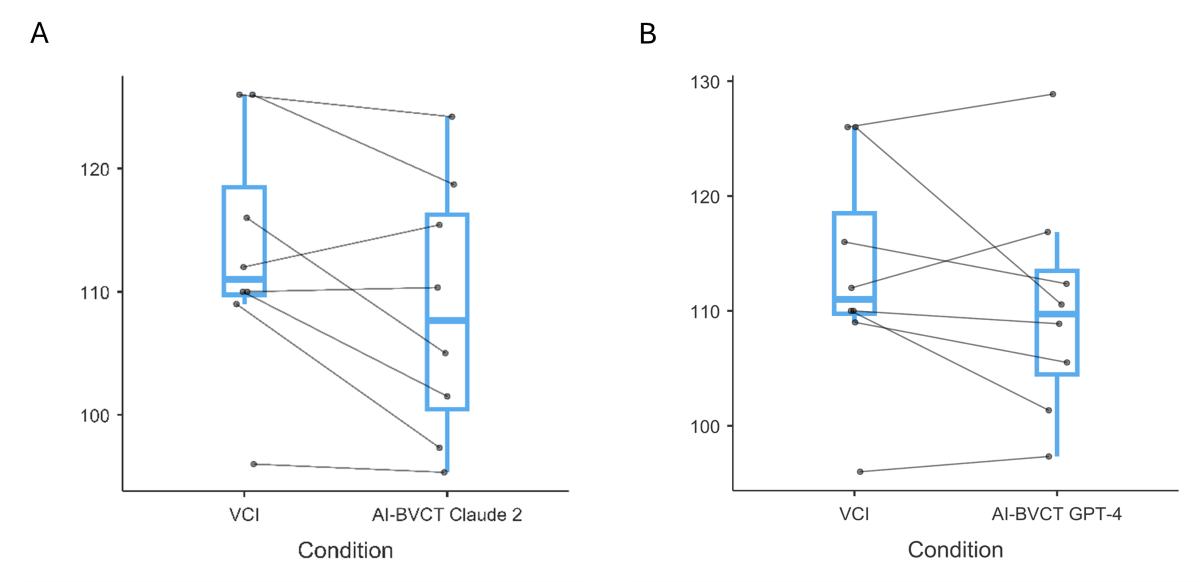
Comparison of VCI scores with AI-BVCT scores from two language models (Claude and GPT-4) in Hebrew-speaking university students (N=8), Israel, September 2023, demonstrating score equivalence within predetermined bounds (SD 15 points). AI-BVCT: artificial intelligence-based verbal comprehension tests.

We hypothesized that the differences between the AI-BVCTs and VCI scores, if any, would be bounded within equivalence bounds of 15 points (half an SD of an IQ score), and examined it using a TOST paired-samples *t* test (Figure 3).

No difference was found between Claude 2 AI-BVCT scores (108.48, SD 10.43) and the VCI score (113.12, SD 9.78; *t*_7_=–2.31, *P*=.05). The mean difference between the VCI and AI-BVCT scores was –4.64, with a 90% CI of –10.65 to 1.37. The 2 one-sided tests were statistically significant, with the score difference significantly higher than the lower bound of –15 (*t*_7_=5.16, *P*<.001), and the score difference significantly lower than the upper bound of 15 (*t*_7_=–9.78, *P*<.001).

No difference was found between the GPT-4 AI-BVCT scores (110.21, SD 9.75) and the VCI score (113.12, SD 9.78; *t*_7_=–1.24, *P*=.25). The mean difference between the VCI and AI-BVCT scores was –2.91, with 90% CI of –9.91 to 4.09. The 2 one-sided tests were statistically significant, with the score difference significantly higher than the lower bound of –15 (*t*_7_= 5.17, *P*<.001), and the score difference significantly lower than the upper bound of 15 (*t*_7_=–7.66, *P*<.001).

In conclusion, the IQ scores derived from the AI-BVCTs can be seen as equivalent on average to the VCI IQ scores.

Next, we examined whether the scores of the AI-BVCTs were within the range of the 95% WAIS CIs (Table 1). 5 out of 8 AI-BVCT Claude 2 scores (62.5%) and 75% of AI-BVCT GPT-4 scores were within the confidence intervals. The 2 proportions were significantly different (z=–0.53, *P*=.59).

**Table 1:** Comparison of individual VCIs^a^ with artificial intelligence–generated test scores: analysis of score agreement within WAIS^b^ CIs in Hebrew-Speaking University Students (N=8) from Northern Israel, September 2023.

	VCI score	WAIS (95% CI)	AI-BVCT^c^ GPT-4 scores		AI-BVCT Claude 2 scores	
			Scores	X^d^ or V^e^	Scores	X or V
1	126	119-131	110.55	X	118.70	X
2	109	103-114	105.51	V	97.32	X
3	112	106-117	116.86	V	115.43	V
4	100	94-106	101.34	V	101.50	V
5	116	110-121	112.35	X	105.00	X
6	110	104-115	108.87	V	110.34	V
7	126	119-131	128.86	V	124.21	V
8	96	91-102	97.32	V	95.33	V
Total	—^f^	—	—	6/8 (75%)	—	5/8 (62.5%)

^a^VCI: verbal comprehension index.

^b^WAIS: Wechsler Adult Intelligence Scale.

^c^AI-BVCT: artificial intelligence–based verbal comprehension tests.

^d^X: Score outside the WAIS 95% CI.

^e^V: Score within the WAIS 95% CI.

^f^Not applicable.

### Differences in AI-BVCT Scores Between the LLMs

**Figure 4:**
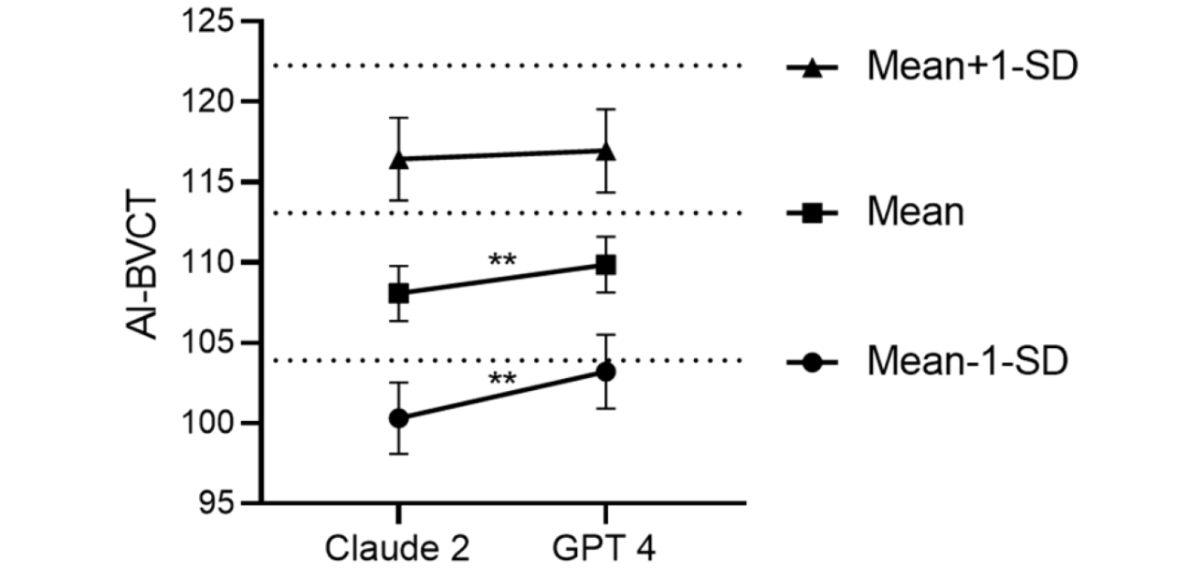
Differential performance of language models (Claude vs GPT-4) in VCI estimation across intelligence levels in Hebrew-speaking university students (N=8), Israel, September 2023. GPT-4 demonstrated superior estimation accuracy at both -1 SD below mean and mean levels. AI-BVCT: artificial intelligence-based verbal comprehension tests. **p<0.001.

Based on these findings, we examined the differences between the 2 LLMs and their interaction with the VCI score using a generalized mixed model (Figure 4). The 2 LLM scores were significantly different (*χ*²_1_=7.2, *P*<.001), with AI-BVCT GPT-4 having a higher mean score (109.86, SD 1.73) than AI-BVCT Claude 2 (108.08, SD 1.71). The difference between the LLMs interacted with the VCI score (*χ*²_1_=3.9, *P*=.04), so at 1 SD below the mean (103.90), GPT-4 (103.18, SD 2.28) estimated the AI-BVCT score significantly higher than Claude 2 (100.31, SD 2.21; *z*=–3.08, *P*=.01). At the mean (113.08), GPT-4 (109.86, SD 1.74) estimated the AI-BVCT score significantly higher than Claude 2 (108.07, SD 1.72; *z*=–2.68, *P*=.01) but at 1 SD above the mean (122.27), GPT-4 (116.97, SD 2.58) was not significantly different than Claude 2 (116.44, SD 2.57; *z*=–0.53, *P*=.59).

These results suggest that Claude 2 underestimated and GPT-4 more closely estimated the true VCI scores, except for the higher VCI scores, where no difference was found between the LLMs. Both LLMs underestimated the true VCI scores at the mean and 1 SD above the mean levels. These results should be interpreted cautiously because the sample size was small and individuals with lower IQ than the mean were underrepresented.

## Discussion

### Principal Findings

The present proof of concept study successfully demonstrated the potential feasibility of using LLMs to generate AI-based verbal comprehension tests. While the sample size was small, as appropriate for an initial feasibility investigation, the strong agreement between AI-BVCT and VCI scores provides compelling preliminary evidence that LLMs can generate psychologically meaningful assessments. However, it is important to note that these findings are based on a student population with above-average intelligence, and the application of this approach to broader populations requires careful consideration. The cognitive profile of university students differs systematically from the general population, particularly from clinical populations or individuals with below-average intelligence, and these differences might affect how LLMs interpret and score responses.

The findings showed a high ICC between the AI-BVCT and the VCI scores, indicating that the AI-generated test effectively discriminated between individuals with different verbal intelligence levels, ranking them similarly to the VCI. A paired-sample *t* test found no significant differences between the AI-BVCT scores and the VCI scores, demonstrating that the AI-generated test was properly calibrated to yield scores comparable to the traditional VCI. Finally, AI-BVCT scores fell within the 95% CI of the VCI scores, providing additional evidence that the AI-generated scores were consistent with what would be expected based on the well-established VCI measure. These preliminary results suggest that LLMs show promise in differentiating between individuals with varying verbal intelligence levels and produce scores that appear to correlate with the traditional VCI test. Further research with larger and more diverse samples is needed to fully establish the reliability and validity of this approach.

The findings of the present proof of concept study provide preliminary evidence that LLMs have the potential to contribute to the field of psychodiagnostics. The ability to accurately assess verbal skills is associated with the diagnosis of significant clinical states [6-10,27]. The results suggest that we may be on the cusp of a transformative moment in psychological assessments, a field that has not been substantially affected by technological advancements. The demonstrated capacity of LLMs to “understand” and process language at a high level holds immense promise for the diagnostic process.

Nevertheless, the process of coding and scoring responses still requires significant human involvement. We found that at times LLMs have difficulty with determining whether a particular answer is correct or incorrect and with accurately adding up scores. Specifically, we identified 2 main types of errors in using LLMs for scoring verbal intelligence tests. The primary issue was with summing up the scores accurately, which occurred frequently. In addition, there were a few instances where the LLMs did not provide correct scoring, although these cases were not numerous. These challenges highlight the current limitations of LLMs in performing precise numerical calculations and occasional misinterpretations in scoring criteria. This situation requires a “human-in-the-loop” for supervision and correction. The need for human oversight is crucial in verifying the accuracy of score summations and ensuring the correct application of scoring criteria. In the future, with improvements in prompting techniques or the development of more powerful models, these issues may diminish. Potential improvements could include enhancing the LLMs’ numerical computation abilities, refining the prompts to emphasize careful score tallying, and developing more robust error-checking mechanisms within the AI system. These advancements could significantly reduce the frequency of calculation errors and improve the overall accuracy of automated scoring. At present, human involvement remains essential to ensure the accuracy of the process.

In addition, we observed differences in scoring patterns between Claude 2 and GPT-4, with GPT-4 providing estimates closer to true VCI scores, especially for lower VCI scores. However, both LLMs underestimated scores at the mean and higher levels. While these differences may stem from varying capabilities in handling linguistic nuances and contextual understanding, particularly in Hebrew, the exact reasons remain unclear and may be temporary due to ongoing model updates. This finding highlights the importance of comparative analyses between LLM platforms in AI-based assessments. Future research should investigate these differences with larger, more diverse samples to ensure reliability across platforms. This underscores the need for ongoing monitoring and evaluation in the development of AI-assisted psychological assessment tools.

The preliminary findings of this study have several potential implications for the field of psychodiagnostics. One of the most significant is the potential of AI-based cognitive tests to make assessment processes more widely accessible and affordable. Traditional cognitive assessments are often expensive and require administration by trained psychologists, limiting their availability to a relatively small subset of the population, primarily those with suspected clinical difficulties. In the future AI-based cognitive tests may be easily generated and administered without the need for a psychologist’s involvement, significantly reducing the associated costs. This could make cognitive assessments more readily available to a broader range of individuals, beyond those with clinical indications. The adaptability of AI-based tests could facilitate their translation and adaptation for use in different cultural and linguistic settings, further expanding their reach [15,22,27].

LLMs enable personalized testing tailored to each individual’s unique characteristics and needs. This adaptive approach allows for the dynamic selection of item difficulty based on the examinee’s performance, ensuring that the test is optimally challenging and informative. Such personalized testing can be particularly beneficial in cases that require repeated assessments over time, such as monitoring cognitive decline [34]. By generating novel items for each administration, AI-based tests can minimize practice effects and provide a more accurate measure of an individual’s cognitive abilities.

The flexibility of AI-generated tests opens up new possibilities for expanding our understanding of cognitive functioning across diverse populations. Large-scale modeling of population distributions for various cognitive attributes may eventually enable the creation of customized norms that consider a wide range of factors, beyond age and gender. For example, norms could be tailored to specific learning disabilities, socioeconomic backgrounds, cultural contexts, and more [35]. This level of customization would allow for a more nuanced and comprehensive assessment of an individual’s strengths and weaknesses. As LLMs continue to advance, we will likely see an increasing integration of these technologies into psychological assessment practices, leading to a new era of personalized and data-driven cognitive evaluation.

Despite the many positive implications demonstrated in this preliminary pilot study, consideration must also be given to the potential risks involved. Most significantly, the present pilot demonstrates the capability of LLMs to perform the technical aspects of test administration, such as test generation, coding, and scoring. Other aspects, like evaluating the context created during the assessment, identifying additional factors through clinical observation such as body language, or discerning problem-solving patterns, were not demonstrated in the current study. It remains uncertain whether LLMs will be able to perform these functions in the near future. In other words, the current research showed the ability to arrive at a similar score as a professional assessment but did not seek to identify or predict factors that may resolve or exacerbate difficulties.

### Ethical Implications and Safeguards

The potential integration of AI-based cognitive assessment tools into psychological practice, even at this preliminary stage, raises important ethical considerations. Drawing from established professional guidelines, including those of major psychological associations, several key ethical domains require attention as this technology develops: protection of individual rights, professional oversight, prevention of misuse, concerns regarding epistemic authority, and current technological limitations [36].

First, privacy and data protection must be paramount [37]. While AI-based assessments could increase accessibility, they also create risks of unauthorized data collection. Professional psychological testing committees emphasize the need for (1) secure data handling protocols, (2) clear consent procedures specifically addressing AI involvement, and (3) strict limitations on data access and retention. These foundational requirements would need further development and validation in larger-scale studies.

Second, the risk of commercial misuse must be addressed through appropriate safeguards [38]. Based on our preliminary findings, key protections should include (1) explicit prohibition of covert cognitive profiling, (2) requirements for professional oversight, and (3) clear limitations on acceptable use cases. While these initial recommendations provide a framework, comprehensive protocols would need to be developed through extensive consultation with professional bodies and validation in clinical settings.

Third, professional standards and oversight are essential. Initial recommendations include maintaining clear boundaries between commercial applications and clinical assessment, requiring professional interpretation of results, and establishing guidelines for identifying and mitigating potential harm [39]. Regular consultation with senior clinical and neuropsychological professionals would be crucial in developing these standards as both the technology and its applications evolve.

Fourth, there is significant concern about overreliance on AI systems, which may be regarded as “all-knowing” with their recommendations taken as absolute [22,27]. This relates to the concept of epistemic authority, which is particularly concerning when working with vulnerable populations such as those with mental health challenges [40]. Professional guidelines emphasize that AI systems should serve only as supportive tools, with ultimate judgment residing with qualified clinicians. This requires (1) clear documentation of AI limitations, (2) training for professionals on appropriate AI integration, and (3) maintenance of critical evaluation practices.

Fifth, our findings highlight important limitations in current AI capabilities. While this study demonstrated the potential for automating technical aspects like test administration and scoring, critical clinical functions, such as observing nonverbal behavior, evaluating problem-solving patterns, and understanding broader context — remain beyond AI capabilities. In addition, the use of public AI models raises specific privacy concerns, as these systems are often trained on user data. Any clinical implementation would require dedicated, secure systems with proper privacy safeguards. Professional guidelines emphasize that validation must go beyond simple score comparison to include a comprehensive assessment of clinical utility and potential risks [27].

The implementation of these safeguards requires ongoing collaboration between AI developers, mental health professionals, and regulatory bodies. Regular consultation with senior clinical, neuropsychological, and educational psychologists will be essential for updating and refining these protocols as both AI capabilities and potential risks continue to evolve. Further research and broader professional consultation will be crucial to establish comprehensive ethical guidelines that protect individual rights while allowing beneficial applications of this emerging technology.

### Study Limitations and Further Studies

While this pilot study included only 8 participants, this sample size was appropriate for our primary aim of demonstrating proof of concept. The strong correlation between AI-generated and traditional test scores, even in this small sample, provides compelling initial evidence for the feasibility of this approach. However, as this study was designed to demonstrate basic viability rather than population-level validation, further research with larger and more diverse samples is required to fully establish the viability of AI-generated testing. Moreover, the small sample was relatively homogeneous in education level and did not represent the full variation in the population. The intelligence examined was high, therefore the study did not take into account lower levels of intelligence. This represents range restriction, and it is possible that at lower levels the results would be different. Further research with larger and more diverse samples is required to fully establish the viability of AI-generated testing. Moreover, the study included a normative population, and future research should also examine populations with diverse cognitive disabilities (eg, learning disability). Another limitation is that the study was conducted in Hebrew; future research needs to examine the capabilities of LLMs in other languages as well.

Overall, these findings provide preliminary support for the use of LLMs in generating valid verbal intelligence assessments without reliance on normative data. The personalized approach allowed the creation of an individualized test tailored to the participant's age and gender background. This study was the first to examine the capabilities of AI in cognitive assessment and intelligence testing as a proof of concept. Given the pilot nature of the research, many additional studies are needed to further validate these initial findings in both the general population and in diverse clinical groups. As a preliminary investigation in this domain, the study helps lay the foundation for psychological testing by AI and serves as a proof of concept**.** Yet, considerably more research is required to fully characterize the use of AI for psychological testing across populations. This pilot study alone is insufficient to draw definitive conclusions, underscoring the need for rigorous continued inquiry in this nascent area. The findings expand the existing knowledge regarding what we know about AI in several aspects, including the ability to create a test and diagnose medical and psychological aspects. It represents an important first step in harnessing LLMs for innovative advances in psychological assessment. The ability to automate test development and scoring shows great promise in making testing more efficient, accessible, and personalized. As LLMs continue to evolve in sophistication, their integration can meaningfully augment and potentially transform standard practices in the field.

### Conclusion

This preliminary study explores the potential of LLMs in generating personalized verbal intelligence tests. While our initial findings are promising, they highlight both opportunities and challenges in using AI for psychological assessments. Further research with larger, more diverse samples is necessary to validate these results and address the ethical considerations of integrating AI into psychological testing practices.

## Abbreviations

AI: artificial intelligence
AI-BVCT: artificial intelligence-based verbal comprehension test
CCC: concordance correlation coefficient
GenAI: generative artificial intelligence
ICC: intraclass correlation coefficient
LLM: large language model
LoA: limits of agreement
TOST: Two One-Sided Tests
VCI: verbal comprehension index
WAIS-III: Wechsler Adult Intelligence Scale

## Data Availability

The datasets generated and analyzed during the current study are available from the corresponding author upon reasonable request. Access is restricted due to privacy considerations and ethical requirements regarding participant confidentiality. The institutional ethics committee will review requests for access to anonymized data which may be granted for research purposes that align with the informed consent provided by participants.
